# A multi-network comparative analysis of whole-transcriptome and translatome reveals the effect of high-fat diet on APP/PS1 mice and the intervention with Chinese medicine

**DOI:** 10.3389/fnut.2022.974333

**Published:** 2022-10-24

**Authors:** Wenya Gao, Junyi Zhou, Xinru Gu, Yanyan Zhou, Linna Wang, Nan Si, Xiaorui Fan, Baolin Bian, Hongjie Wang, Haiyu Zhao

**Affiliations:** Institute of Chinese Materia Medica, China Academy of Chinese Medical Sciences, Beijing, China

**Keywords:** Alzheimer’s disease, high-fat diet (HFD), Ribo-seq, RNA-seq, Huanglian Jiedu Decoction

## Abstract

Different studies on the effects of high-fat diet (HFD) on Alzheimer’s disease (AD) pathology have reported conflicting findings. Our previous studies showed HFD could moderate neuroinflammation and had no significant effect on amyloid-β levels or contextual memory on AD mice. To gain more insights into the involvement of HFD, we performed the whole-transcriptome sequencing and ribosome footprints profiling. Combined with competitive endogenous RNA analysis, the transcriptional regulation mechanism of HFD on AD mice was systematically revealed from RNA level. Mmu-miR-450b-3p and mmu-miR-6540-3p might be involved in regulating the expression of *Th* and *Ddc* expression. MiR-551b-5p regulated the expression of a variety of genes including *Slc18a2* and *Igfbp3*. The upregulation of *Pcsk9* expression in HFD intervention on AD mice might be closely related to the increase of cholesterol in brain tissues, while Huanglian Jiedu Decoction significantly downregulated the expression of *Pcsk9*. Our data showed the close connection between the alterations of transcriptome and translatome under the effect of HFD, which emphasized the roles of translational and transcriptional regulation were relatively independent. The profiled molecular responses in current study might be valuable resources for advanced understanding of the mechanisms underlying the effect of HFD on AD.

## Graphical Abstract

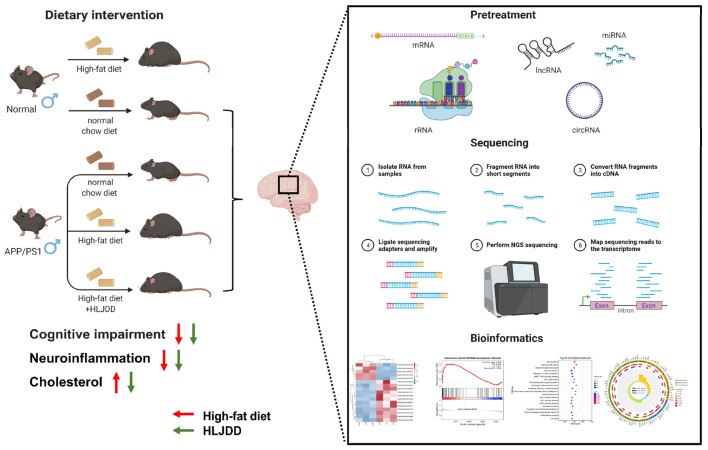

## Introduction

Alzheimer’s disease (AD) was a progressive neurodegenerative disease related to aging, characterized by the pathological hallmarks of extracellular accumulation of amyloid-β (Aβ) plaques and intracellular accumulation of neurofibrillary tangles. AD was caused by the complex interaction of multiple mechanisms, and the etiology was still unclear. AD was generally considered to be related to genetic and environmental factors (1). Diet and nutrition displayed potential for non-pharmacological AD prevention. However, different studies on the effect of high-fat diet (HFD) on AD pathology in AD models reported conflicting conclusions. For instance, the HFD feeding induced Aβ accumulation and cognitive decline in APP/PSEN1 mice. Systemic inflammation and obesity could be reversed by a low-fat diet (2). Aβ and HFD had a synergic effect, leading to the impairment of endoplasmic reticulum and mitochondrial functions, glial reactivity status alteration and inhibition of insulin receptor signaling. These metabolic alterations would favor neuronal malfunction and eventually neuronal death by apoptosis, hence causing cognitive impairment (3). However, other studies found that HFD might promote better cognitive function by improving blood-brain barrier function and attenuating brain atrophy in AD, but it didn’t seem to affect Aβ levels (4–6). Therefore, the influence of HFD on the progression of AD was controversial. A clear understanding of the HFD role in AD pathology would help improve the quality of life and relieve the demand pressure of aging population on the overall resources of society.

Huanglian Jiedu Decoction (HLJDD) was composed of Rhizoma coptidis, Radix scutellariae, Cortex phellodendri, and Fructus gardenia at a ratio of 3:2:2:3. HLJDD was a classic prescription for clearing away heat and toxic materials in past dynasties. Alkaloids, flavonoids and iridoid glycosides were the mainly active ingredients in the prescription (7). Modern research showed that HLJDD had many pharmacological effects, such as anti-inflammatory, antibacterial, antioxidant, lipid-lowering and hypoglycemic, antitumor, neuroprotection and so on (8). The literature researches and previous experiments of our research team showed HLJDD could reduce the accumulation of Aβ and Tau in central of APP/PS1 mice, improve cognitive ability, and ameliorate the lipids and inflammatory environment in the center and periphery (9). Furthermore, HLJDD could regulate the metabolism of central neurotransmitters, amino acids, peripheral bile acids, and relieve AD symptoms in combination with intestinal flora (10). In this study, we would continue to explore the curative effect and mechanism of HLJDD on HFD plus AD model mice.

Genome-wide association studies (GWAS) identified the following genes associated with AD risk: *ABCA7*, *BIN1*, *CASS4, CD33*, *CD2AP*, *CELF1, CLU*, *CR1*, *DSG2, EPHA1*, *FERMT2, HLA-DRB5-DBR1, INPP5D, MS4A*, *MEF2C*, *NME8*, *PICALM*, *PTK2B, SLC24H4 RIN3*, *SORL1*, *ZCWPW1*, *PLD3, and TREM2* (11). The loci identified by large GWAS analysis for late-onset Alzheimer’s disease (LOAD) were related to immune response, inflammation, lipid metabolism, endocytosis/intracellular trafficking, and cell migration (12). APP, PSEN1, and PSEN2 were associated with early onset AD (13), while APOE4 was considered as a risk factor for LOAD (14), in addition to genes related to cholesterol metabolism and immune response that could also increase the risk of LOAD (15). RNA sequencing (RNA-seq) provided an unbiased way to investigate the genome-wide transcriptome profiling, and it could help construct the complicated gene regulatory network in the dynamic progression of human diseases.

Competing endogenous RNAs (ceRNAs) were RNAs in the complex network of transcriptional regulation in organisms, including protein-coding mRNA, long non-coding RNA (lncRNA), pseudogene, and circular RNA (circRNA). The regions of these RNAs could be bound by systematically functionalizing microRNA (miRNA) response element (MRE)-harboring non-coding RNAs. Competing to bind common miRNAs through common MREs, the RNAs interacted and regulated the expression of target gene transcripts. Thus, through the miRNA, these RNAs could interact with each other to form complex miRNA-mediated ceRNA networks. The interaction relationship showed the possible functions of the lncRNAs and circRNAs. Significant changes in lncRNA were also observed in AD models, with studies reporting the upregulation of *MRAK088596, MRAK081790, and MAPK10* and downregulation of *BC092582, MRAK050857, and S100A8* in AD rats (16). CircRNA had been shown to play an important role in the development of AD by affecting neurogenesis and injury, Aβ deposition, neuroinflammation, autophagy and synaptic function through miRNA sponging. Large number of differentially expressed circRNAs were presented in the brains of AD patients (17). The association of various human miRNA with disease had been experimentally validated. A large set of miRNA-mRNA associations that were found in AD patients (18) and played important roles in the regulation of Aβ precursor protein expression, lytic enzyme activity and APP pathway-related signaling molecules. They also regulated tau protein expression, tau phosphorylation-related kinase and phosphatase function. The study showed that a decrease in miR-29a/b could contribute to increased BACE1 and Aβ levels in sporadic AD (19). MiRNAs also had the effect on learning and memory processes, regulating L-LTP, excitatory glutamatergic systems and other synaptic transport (20).

Gene expression in currently studies was mainly at the transcriptional level, largely ignoring translational regulation. However, translation regulation was accounted for more than half of all regulation in biological genetic information transfer and was the most important form of regulation in the cells. Ribosome profiling (Ribo-seq), in which next-generation sequencing used to identify ribosome-protected mRNA fragments, thereby revealing the positions of the full set of ribosomes engaged in translation, has emerged as a transformative technique for enabling global analyses of *in vivo* translation and coupled, translational events (21). Ribo-seq had been widely used in different species (22–25). The researchers analyzed gene expression in cerebral cortex of two AD model mouse strains, CVN (APP_Sw_DI/NOS2^–^*^/^*^–^) and Tg2576 (APP_*Sw*_), by tandem RNA-seq and Ribo-seq. AD model mice had similar levels of transcriptome regulation, but differences in translatome regulation (26).

Previously, we detected that long-term HFD intervention altered the levels of cholesterol and polyunsaturated fatty acids in the brain tissue of APP/PS1 mice and influenced the secretion of peripheral bile acids (10). Translational regulation was considered to play a vital role in gene expression, but whether HFD functions through the regulation of gene translational level was still unclear. The mechanism linking HFD in the regulation of transcriptome and translatome in APP/PS1 mice had not yet been systematically elucidated. In order to analyze the overall effects of HFD on the AD mice, whole-transcriptome sequencing (mRNA-seq, lncRNA-seq, circRNA-seq, and miRNA-seq) and Ribo-seq were used to explore. In addition, the associations between transcriptional and translational levels corresponding to this phenotype further screened out some known target genes and new functional genes, followed by functional interaction prediction analysis. In summary, our analysis could reveal distinct roles of translational and transcriptional regulation in HFD intervention on AD mice. This study aimed to provide a new direction for the treatment of AD through the joint analysis of transcriptome and translatome.

## Materials and methods

### Animal and diet

Five-month-old SPF grade male C57BL/6J-TgN (APP/PS1) transgenic mice and C57BL/6J wild type mice (Shanghai Model Organisms Co., Ltd., Production license number SCXK 2014-004) were used in this study. All experiments and animal care in this study were conducted in accordance with the National Institutes of Health Guide for the Care and Use of Laboratory Animals (NIH Publications No. 8023, revised 1978) and the Provision and General Recommendation of Chinese Experimental Animals Administration Legislation. The study was approved by the Institutional Animal Care and Use Committee of the Beijing animal science Co., Ltd., and the animal ethics approval number was IACUC-2018100605. Animals were housed in a single cage with chow and water *ad libitum* and a 12 h light-dark cycle and kept under a consistent temperature of 21°C. Wild type mice were assigned to the normal group (the Nor group, fed a normal chow diet) and the Nor_HFD group (fed a HFD diet). The HFD diet contained 63.6% basic feed, 15% lard, 20% sucrose, 1.2% cholesterol, and 0.2% cholate (Beijing Keao Xieli Feed Co., Ltd.). APP/PS1 mice were randomly allocated into 3 groups: one was fed a normal chow diet (the AD group), and one was fed a HFD diet (the AD_HFD group), and another was fed HFD diet and the powder of HLJDD (the H_H group). The HLJDD powder was prepared in our laboratory as previously described (7). Our research was a preventive protocol, and the gavage dose was 344 mg/kg/d (HLJDD) for 3 months. Animal weights were recorded every week.

### Morris water maze test

The Morris water maze (MWM) test was performed to detect spatial memory as previously described with a slight modification (27). Mice participated in a navigation test for four consecutive days. Four sequential training trials began by placing the animals facing the wall of the pool but changing the drop position for each trial. If the mouse found the platform before the 90 s cut-off, allowing the mouse to stay on the platform for 10 s then return it to its home cage. Otherwise, we placed the mouse on the platform and allowed it to stay there for 20 s. The mouse was trained in different direction. We repeated the training for all mice in the trail in the next 4 days. In probe trial, we removed the platform from the pool and the test time was 60 s. Escape latencies, time spent or distance traveled in the target quadrant and platform-crossing times were recorded and analyzed using the analysis management system (Beijing Zhongshi Kechuang Co., Ltd.).

### Brain sample collection

After the MWM test, all mice rested for 4 days under normal conditions. After anesthesia with 10% chloral hydrate, serum was collected from the heart, followed by removal of brain tissue on a sterile table, rinsing with pre-cooled RNAse-free saline at 4°C, blotting up. Then put the sample into 1.5 mL labeled RNase-free EP tubes, which were rapidly frozen in liquid nitrogen for 30 min and stored at −80°C in the refrigerator until use. Whole brain had been ground in liquid nitrogen.

### Western blot assay

Western blot (WB) analysis for brain tissues were lysed in precooled RIPA buffer with the protease inhibitor PMSF (Amresco), and protein concentrations were determined using a BCA protein assay kit. Protein samples were separated on 12% sodium dodecyl sulfate polyacrylamide gels electrophoresis (SDS-PAGE) and transferred onto NC membranes. Then, membranes were blocked in 5% non-fat milk for 30 min at room temperature and incubated with primary antibodies overnight at 4°C. Membranes were then washed and incubated with HRP conjugated goat anti-rabbit and HRP-conjugated goat anti mouse (1:10,000) secondary antibodies for 40 min at room temperature followed by development using ECL detection. The obtained bands were then scanned and analyzed using ImageJ software, and band density was assessed using Total Lab Quant V11.5 (Newcastle upon Tyne, United Kingdom).

### RT-PCR

Total RNA was extracted from brain tissue using TRIzol reagent (ELK Biotechnology, China) according to the manufacturer’s instructions. RNA concentrations were equalized and converted to cDNA using the EntiLink™ 1st Strand cDNA Synthesis Kit (ELK Biotechnology, China). Gene expression was measured using a StepOne™ Real-Time PCR system. The sequences of primers used in these experiments were listed in the Supplementary material.

### RNA-seq and Ribo-seq

The experimental procedure and data analysis were listed in the Supplementary material.

### Data availability

The datasets presented in this study can be found in online repositories. The names of the repository/repositories and accession number(s) can be found here: http://bigd.big.ac.cn/gsa/, CRA007307.

### Statistical analysis

The results were expressed as the mean ± standard error of the mean (SEM). The significance of difference among the groups was assessed by Student’s *t*-test for two groups and one-way ANOVA for more than two groups, followed by the LSD and Games-Howell post-test. Statistical calculations were performed using SPSS 20 software. Differences with statistical significance were denoted by *P*-value less than 0.05.

## Results

### Evaluation of high-fat diet intervention in the APP/PS1 mice

**FIGURE 1:**
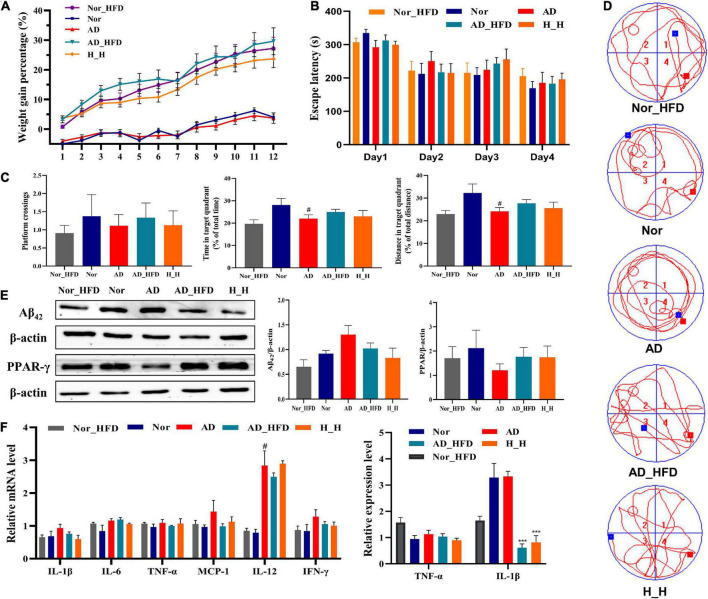
The effect of HFD on APP/PS1 mice cognitive impairment. **(A)** The weight gain percentage with normal diet or HFD for 3 months, respectively. **(B)** Escape latency during spatial acquisition training. **(C)** The platform crossing number, distance traveled percentage in the target quadrant and time spent percentage in the target quadrant in the spatial probe test (*n* = 10). **(D)** The real-time monitoring of mice motion track Morris Water Maze test experiment. **(E)** The relative protein concentration of Aβ_42_ and PPAR-γ detected by WB (*n* = 3). **(F)** The expression of inflammatory cytokine detected by RT-PCR and Elisa method in brain tissue. All the results are expressed as the mean ± SEM; ^#^*P* < 0.05, ^##^*P* < 0.01, ^###^*P* < 0.001 (compared to the Nor group); *P < 0.05, **P < 0.01, ***P < 0.001 (compared to the AD group).

After 3 months of HFD administration, there was an obvious weight gain in AD_HFD mice at a rapid pace compared to normal chow diet mice (Figure 1A). HFD significantly accelerated the percentage of weight gain, and HLJDD could slow down the pace of weight gain caused by HFD. In the MWM test, the sequential changes in the average escape latency during spatial acquisition training were shown in Figure 1B. With the increase of training times, the incubation period of each group gradually was shortened. AD mice displayed longer average escape latency compared to Nor mice on day 2, 3, and 4. AD_HFD and H_H mice were not exhibited significantly different compared to AD mice. In the spatial probe test, the platform crossing number in AD mice was lower compared to the Nor group. Meanwhile, the percent distance and time spent in the target quadrant were significantly lower than those in the Nor group (*P* < 0.05). The platform crossing number, percent distance and time spent in the target quadrant in the AD_HFD group were higher than those in the AD group, indicating that it had improvement tendency toward the cognitive impairment with the HFD intervention. There is no significant difference between the H_H group and AD group (Figure 1C). The trajectory map of AD mice was disorganized and purposeless (Figure 1D). WB analysis revealed that the levels of Aβ_42_ were increased in AD group compared to the Nor group and decreased in the AD_HFD and H_H groups compared with the AD group. The levels of PPAR-γ were decreased in AD mice compared to the Nor group, while increased in AD_HFD and H_H groups compared with AD group (Figure 1E). The mRNA expression of different proinflammatory cytokines, *IL-1*β, *IL-6, TNF*-α, *MCP-1*, *IL-12A, IL-12B*, and *IFN*-γ, were upregulated in the AD group compared to the Nor group. The mRNA levels of proinflammatory cytokines were slightly reduced in response to the HFD intervention. Inflammatory cytokine levels were also detected by enzyme-linked immunosorbent assay (ELISA). The expression of TNF-α and IL-1β was decreased in the AD_HFD and H_H groups compared to the Nor group, and IL-1β levels was significantly reduced (*P* < 0.001) (Figure 1F). Taken together, the HFD intervention might have the effects of relieving inflammation in AD model mice.

### Overview data of mRNA-seq and Ribo-seq

**FIGURE 2:**
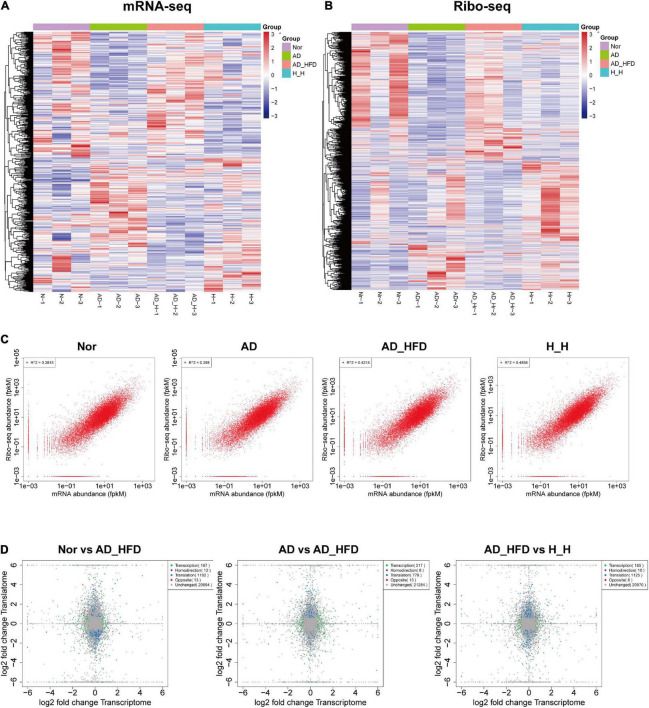
Overview of genes identified by transcriptome and translatome. Heatmap of cluster analysis of DEGs in the transcriptome **(A)** and translatome **(B)**. N-1, N-2, and N-3 represent the Nor group biological repetition. AD-1, AD-2, and AD-3 represent the AD group biological repetition. AD_H-1, AD_H-2, and AD_H-3 represent the AD_HFD group biological repetition. Blue represents the lowest and red represents the highest. **(C)** Scatter plot of transcriptome and translatome in four groups. **(D)** Quadrant diagram of the fold change at transcriptional and translational levels.

At the mRNA sequence profiling, 18490 detected genes were identified. At the Ribo sequence profiling, 17433 detected genes were identified. The gene expression levels for both the transcriptome and the translatome were similar with normal distribution. The distribution of expression abundance among the samples was shown in Supplementary Figures 1A,B. The peaks of the samples were generally consistent, indicating that there was little difference in the overall expression of the genes at the transcriptional and translational levels among the samples. The heat maps were shown in Figures 2A,B. Pearson correlation coefficient (R) between Ribo sequence abundance and mRNA abundance was calculated, and the scatter plots (Figure 2C) were drawn to analyze the correlation at the translational and transcriptional levels. The *R*-values of Nor, AD, AD_HFD, and H_H groups were 0.62, 0.63, 0.65, and 0.7, indicating a moderate correlation between mRNA abundance and Ribo sequence abundance in four groups. Principal component analysis (PCA) of mRNA-seq and Ribo-seq were shown in Supplementary Figures 1C,D. The ribosome-protected fragments (RPFs) length distribution peaked at 28 nt in both groups (Supplementary Figure 1E). The mRNAs protein-coding sequences (CDS) contained the majority of RPFs in four groups, with an average distribution ratio of 89.26, 88.03, 88.02, and 87.95%, for the Nor, AD, AD_HFD and H_H groups separately. The 5′ UTR and 3′ UTR distribution ratio was less than 3%, respectively (Supplementary Figure 1F). These data demonstrated the reproducibility and reliability of this analysis. The identification and quantification information for the transcriptome and translatome were shown in Supplementary Tables 2, 3.

### Differential transcriptome analysis

#### Analysis of differently expressed genes in Alzheimer’s disease mice with high-fat diet

**FIGURE 3:**
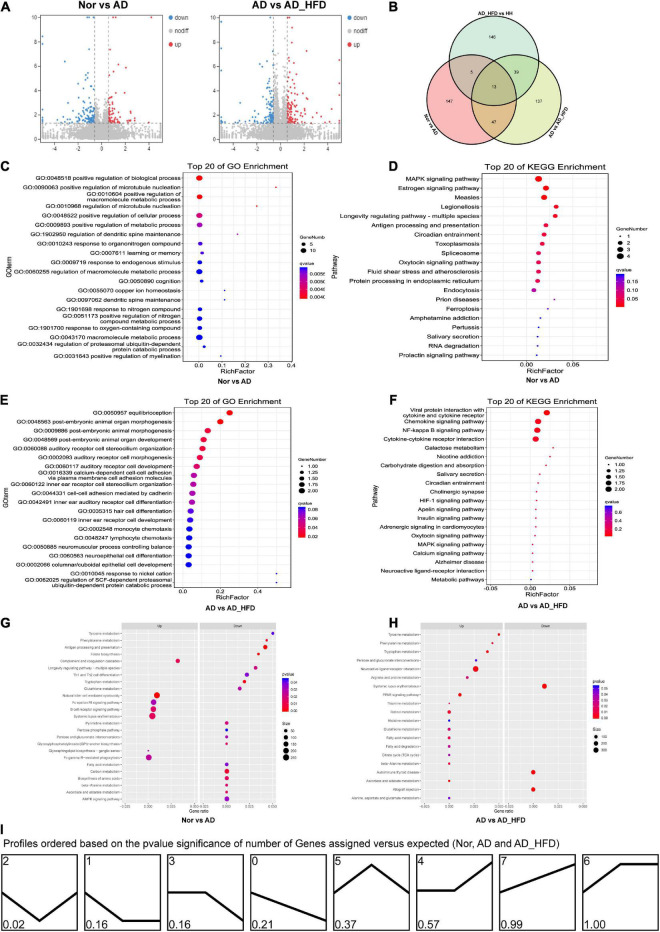
Transcriptome analysis. **(A)** Volcano plots of differentially expressed mRNA between groups in transcriptome. **(B)** Venn diagram showing the distinct and overlapping differential genes between groups of the transcriptome. **(C)** GO enrichment analysis of the transcriptome between the Nor and AD groups. **(D)** KEGG pathway analysis of the transcriptome between the Nor and AD groups. **(E)** GO enrichment analysis of the transcriptome between the AD and AD_HFD groups. **(F)** KEGG pathway analysis of the transcriptome between the AD and AD_HFD groups. **(G)** GSEA of the transcriptome between the AD and AD_HFD groups. **(H)** GSEA of the transcriptome between the AD and AD_HFD groups. **(I)** Trends in genetic variation among the Nor, AD and AD_HFD groups.

Based on the HISAT2 comparison results, we reconstructed the transcripts using StringTie and calculated the expression of all genes in each sample. Using the reads count data of gene expression levels of each sample, we analyzed the difference between groups using DESeq2 software with *P* < 0.05 and | log_2_FC| ≥ 0.585 as significant differentially expressed genes (DEGs). 87 genes were up-regulated and 125 genes were down-regulated in the AD group compared to the Nor group. Compared to the AD group, 116 and 120 genes were up- and down-regulated in the AD_HFD group, respectively. 30 genes were significantly differentially expressed in both AD and AD_HFD groups compared to the Nor group. 13 of them were reduced and 16 were significantly increased in AD mice. In addition, *Gpr151* was reduced in the AD group but increased in the AD_HFD group. The volcano plot and Wayne plot of differentially expressed genes between the groups were shown in Figures 3A,B.

KEGG analysis (Nor vs. AD group) identified the significant enrichment pathways including apoptosis, MAPK signaling pathway, neuroactive ligand-receptor interaction, purine metabolism, dopaminergic synapse, serotonergic synapses, etc. The AD and AD_HFD groups were significantly enriched in the KEGG at dopaminergic synapse, synaptic vesicle cycle, cholinergic synapse, estrogen signaling pathway, neuroactive ligand-receptor interactions, MAPK signaling pathway, TNF signaling pathway, galactose metabolism, serotonergic synapse, starch and sucrose metabolism (Figures 3D,F). DEGs among Nor, AD and AD_HFD groups found by KEGG enrichment analysis were focused on the regulation of a variety of synapses, including dopamine, choline and serotonin, which indicated that the expression levels of regulatory neurotransmitter genes were altered in the brain tissue of AD mice and HFD intervention also had a greater effect on these genes.

The results of GO enrichment analysis results were shown in Figures 3C,E. The differential genes in the brain tissue of normal and AD mice were mainly enriched in behavioral, nervous system, neurotransmitter, immune and chemotactic terms in biological process ontology. The result suggested that neurotransmitter metabolism and inflammatory responses were disturbed in the brain tissue of AD mice compared to normal mice, and HFD intervention could affect neurotransmitter metabolism and inflammatory responses in AD mice.

*Dusp1, Gpr151, Th, Ddc*, and *Npas4* were upregulated, while *Ccl21b* and *Slc1a1* were downregulated in the AD_HFD group compared with the AD group. Dual specific phosphatase (DUSP) played an important immunomodulatory function through the DUSP-MAPK phosphatase pathway (28). DUSP1 played an important negative regulatory role in the inflammatory immune response of macrophages induced by Toll-like receptor ligand stimulation (29). Increased *Dusp1* expression in the HFD group suggested a close association with partial remission of inflammation in brain tissue. Tyrosine hydroxylase (TH) was a catecholamine rate-limiting enzyme that catalyzed the conversion of tyrosine to dihydroxyphenylalanine and regulated the production of dopamine, noradrenaline and epinephrine neurotransmitters. Aromatic L amino acid decarboxylase (DDC) catalyzed the conversion of dopa to dopamine. Both were more highly expressed under HFD, indicating that HFD intervention would strengthen the dopamine neurotransmitter synthesis in the brain tissue of AD mice. Neuronal PAS domain binding protein 4 (NPAS4) mRNA was upregulated in the AD_HFD group, suggesting that HFD could enhance the regulation of glutamatergic and GABAergic synapses. In addition, GPR151 was associated with pineal synaptic function and nicotinic uptake.

##### GSEA analysis

In order to further understand the effect of HFD in AD, GSEA analysis was performed. GSEA analysis of KEGG revealed that 94 of the 324 gene sets were upregulated in the AD group compared to Nor group, including glycosphingolipid biosynthesis-ganglio series; 9 gene sets were downregulated in the AD group, including the specific pathways of folate biosynthesis, antigen processing and presentation, phenylalanine metabolism, tryptophan metabolism, alanine metabolism, ascorbate and aldarate metabolism, DNA replication, GPI-anchor biosynthesis, and carbon metabolism. 170 gene sets were upregulated in the AD_HFD group compared to AD group, while 154 gene sets were downregulated in the AD_HFD group. The results of the GSEA analysis with *P* < 0.05 were shown in Figures 3G,H and Supplementary Figure 2.

Differential genes in AD patients were mainly enriched in immune and metabolic pathways (30). Meta-analysis found the levels of acetylcholine and GABA were significantly lower and the levels of glycine were slightly higher in the cerebrospinal fluid of AD patients. Meanwhile, anaerobic glycolysis and the pentose phosphate pathway and the tricarboxylic acid cycle pathway were enhanced as well (31). Methionine, tryptophan and tyrosine purine metabolic pathways were altered in mild cognitive impairment (MCI) and AD patients (32). Phenylalanine, tyrosine and tryptophan levels were reduced in the serum of AD patients (33). Analysis of mRNA expression levels showed the downregulation of multiple metabolic pathways in AD mice, including phenylalanine, tryptophan and alanine metabolism. The effect of HFD on metabolism in AD mice was more extensive, with 18 of the 23 significantly upregulated gene sets being related to metabolic pathways, mainly involving the metabolism of amino acids and carbohydrates. Compared to normal mice, AD mice had metabolic abnormalities in lipid and amino acid metabolism. The metabolic pathways of phenylalanine, tryptophan and alanine were down-regulated in the AD group, while significantly up-regulated in the AD_HFD group, suggesting that HFD could regulate amino acid metabolism in the brain tissue of AD mice. The AD_HFD group could modulate the ascorbate and aldehyde metabolism pathways. In addition to substance metabolism, HFD also up-regulated PPAR signaling pathway and neuroactive ligand-receptor interactions pathway.

##### Differential gene expression trend analysis

A total of 8 patterns of gene trends among the Nor, AD and AD_HFD groups were plotted (Figure 3I), with profile 2 being significant and containing 72 genes. The analysis of the trends suggested that HFD intervention could callback profile2 genes, which might be associated with moderating the process in AD pathology. The profile2 genes were significantly enriched in KEGG pathways such as cholinergic synapses, dopaminergic synapses, MAPK signaling pathways, synaptic vesicle recycling, purine metabolism, serotonergic synapses, etc. The involved genes were *Slc6a3*, *Chrnb4*, *Fos*, *Hspa1b*, *Igfbp3*, *Gm45837*, *Dusp1*, *Slc18a2*, *Hspa1a*, *Itk*, *Gucy2c*, *Wnt9b*, and *Chrna6*.

*Slc6a3* encoded the dopamine transporter and its variant carriers reduced cognitive performance and were at greater risk of developing dementia (34). In mouse models the activation of the endogenous *Nlrp3* promoter was catalyzed only by the dopaminergic neuron specific *Slc6a3* promoter. Dopaminergic neurons could accumulate NLRP3 inflammatory activators such as reactive oxygen species, dopamine metabolites, and misfolded proteins along with organismal aging. Activation of NLRP3 could induce inflammation and improve the cognitive impairment during normal aging and neuropathological processes (35). Heat shock protein (HSP) protected cells from oxidative stress, while HSP70 inhibited tau protein aggregation (36), effectively treating AD types with aging-related conditions (37). The expression of mRNA encoding HSP70 was increased in AD patients (38, 39), and APMAP levels were reduced. Nevertheless, HSPA1A and CD-M6PR levels, which controlled Aβ production, were increased (40). Proteomic studies found that HSPA1A levels in cerebrospinal fluid extracellular vesicles could monitor the course of AD (41). HSPA1B was associated with non-cognitive alterations in AD, and HSPA1B genes had significant AD non-cognitive symptoms (42). ITK regulated the signaling network downstream of T cell receptor signaling and influenced the differentiation of effector T cells. *Itk* could promote autoimmunity and central nervous system (CNS) inflammation (43). Suppression or deletion of *Itk* resulted in a decrease in Tr1 and TH17 cells and an increase in Treg cells (44).

#### Analysis of differently expressed genes in Alzheimer’s disease mice combined with Huanglian Jiedu Decoction and high-fat diet

In comparison with the AD_HFD group, the level of 95 genes were up-regulated and 108 genes were down-regulated after HLJDD administration (Supplementary Figure 3A). 52 genes were significantly changed among the AD, AD_HFD and H_H groups, of which 26 were decreased and 26 were increased in the AD_HFD group, while in the H_H group the gene levels were back-regulated. A total of 27 genes were significantly altered among the Nor, AD_HFD and H_H groups, of which 17 were reduced and 9 were increased in the AD_HFD group, while the H_H group significantly modulated the changes of these genes.

The AD_HFD and H_H groups were significantly enriched in neuroactive ligand-receptor interaction, tyrosine metabolism, folate biosynthesis, galactose metabolism, Th1 and Th2 cell differentiation, etc. Term was mainly enriched in the GO database for nervous system, behavior and neurotransmitters (Supplementary Figures 3B,C).

GSEA analysis of the KEGG pathway revealed that 136 of the 324 gene sets were upregulated in the H_H group compared to the AD_HFD group, involved the oxidative phosphorylation pathway. GSEA analysis of genes that changed between the two groups were shown in Supplementary Figure 3D. HLJDD up-regulated carbohydrate digestion and absorption, the phospholipase D signaling pathway, the longevity regulation pathway and axon regeneration, and down-regulated tyrosine metabolism, neuroactive ligand-receptor interactions, Th17 cell differentiation, the IL-17 signaling pathway, cholesterol metabolism and MAPK signaling pathway. The transcriptional level also indicated that HLJDD could inhibit the inflammatory response and regulate lipid metabolism, in addition to suggesting a regulatory effect on neuronal regeneration and neurotransmitter-like metabolism.

No significant changes were found in the trend analysis among the AD, AD_HFD and H_H groups. Profile 2 and 3 had significant changes in the Nor, AD_HFD and H_H groups (Supplementary Figure 3E), with 83 and 68 genes, respectively. Profile 2 genes were significantly enriched in homologous recombination, non-homologous end splicing, RIG-I-like receptor signaling pathway, NOD-like receptor signaling pathway and retinol metabolism, involving *Mre11a*, *Tbkbp1*, *Irf3*, *Gbp5*, *Rpe65*, *Gm5136*, and *Rdh16*. Genes in profile 3 were significantly enriched in neuroactive ligand-receptor interactions, folate biosynthesis, tyrosine metabolism, mitochondrial autophagy, cAMP signaling pathway, involving *Htr1f*, *Th*, *Alpl*, *Adh7*, *Prkn*, *Xrcc5*, *Nmu*, *Fosb*, *Atg9b*, *Drd1*, *Calca*, *Adora2a*, *Npffr2*, and *Trh*.

#### Analysis of genes related to cholesterol metabolism in different intervention methods

**FIGURE 4:**
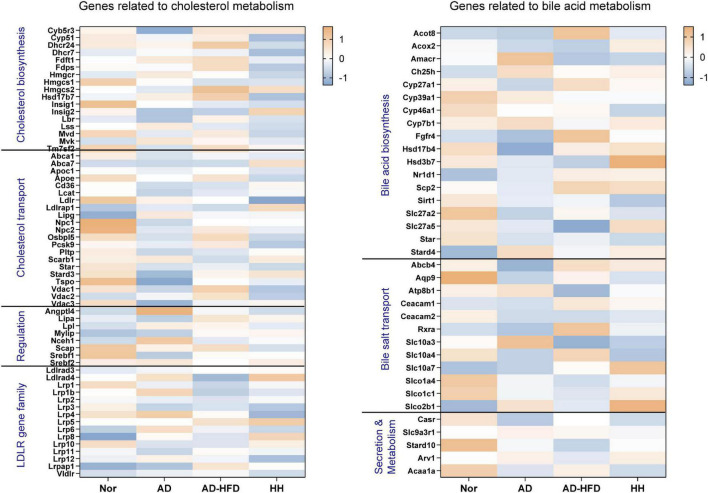
Heatmap of cholesterol and bile acid-related genes in the transcriptome. Blue represents the lowest while orange represents the highest.

Using the transcriptome sequencing data as a benchmark, genes related to cholesterol transport, cholesterol biosynthesis, low density lipoprotein receptor (LDLR) gene family, bile acid biosynthesis, transport, secretion and metabolism were screened (Figure 4). Low expression genes were filtered out. The expression level between groups for the gene sets were compared, combined with the previous quantitative results, the effect of HFD on gene expression in the brain tissue of APP/PS1 mice was further analyzed.

Analysis of the above screened genes revealed that *Pcsk9*, a cholesterol transport-related gene, was significantly decreased in the H_H group. PCSK9 promoted low density lipoprotein (LDL) degradation. The upregulation of *Pcsk9* expression in the AD_HFD group might be closely related to the increase in cholesterol in brain tissues, while HLJDD significantly downregulated *Pcsk9* expression. The expression of *Slc10a4*, a bile acid transport-related gene, was significantly reduced in the AD group and significantly increased in the AD_HFD group and significantly reduced in the H_H group. SLC10A4 was a family of bile acid sodium cotransport proteins that were activated by proteases to transport bile acids (45) and could be involved in the transport of bile acids in brain tissue. SLC10A4 was significantly reduced in brain tissue at highly phosphorylated tau protein lesions, suggesting its close association with AD pathology (46). CYP27A1 regulated the synthesis of primary bile acids in the alternative pathway, and the results of our previous experiments on serum bile acids in mice also showed that HFD intervention increased the level of CDCA produced by the alternative pathway, once again confirming that HFD intervention could cause a significant increase in bile acid synthesis in AD mice.

### Structural analysis of transcripts

The main variants type of single nucleotide polymorphism (SNP) was non-synonymous SNV, while the main variants location of SNP was in intronic. The SNP mutation types were transition (80.22%) and transversion (19.78%). A->G in transition accounted for the largest proportion. G->T in transversion accounted for the largest proportion. Among the analysis of alternative splicing, skipped exon accounted for the most in four groups (Supplementary Figure 4).

### Differential translatome analysis

Differential translation genes (DTGs) between groups were performed using edgeR software. Compared to the Nor group, 336 DTGs were significantly up-regulated and 881 DTGs were down-regulated in the AD group; 603 and 194 DTGs were significantly up- and down-regulated in the AD_HFD group compared to the AD group, respectively; while H_H resulted in 851 and 292 DTGs being significantly up- and down-regulated, respectively.

A total of 382 differentially translated genes were co-varied among the Nor, AD and AD_HFD groups, of which 59 were up-regulated and 323 down-regulated in the AD group, while the HFD intervention significantly back-regulated changes in translated gene expression in the AD group. A total of 201 differentially translated genes were co-varied among AD, AD_HFD and H_H groups, 90 translated genes were down-regulated and 111 translated genes were up-regulated in the AD_HFD group, respectively. Except for *Zbtb16* and *Tmem121b*, all genes were significantly modulated by HLJDD.

### Joint analysis of transcription and translation

#### Analysis of differentially expressed genes and differential translation genes

There were 212 DEGs and 1217 DTGs between the Nor and AD groups, and 25 genes that changed at both levels. The combination of transcriptional and translational analysis revealed that *Sgk1*, *Myo1f*, *Oip5*, and *Cst7* were both up-regulation; *Iqschfp*, *Gm45837*, *Itga2b*, *Alb*, *Npas4*, *Fos*, *Ccn1*, and *Dusp1* were both down-regulation; *Npy, Ptchd4, Clcc1, Thbs4*, and *Cdh12* were up-regulation in transcriptome and down-regulation in translatome; *Grid2ip, Gucy2c, Th, Eva1a, Ngb, Slc10a4, Hs3st3b1, and Hspb1* were down-regulation in transcriptome and up-regulation in translatome. Homodirectional genes were enriched in learning, memory, cognition, regulation of cell death, response to lipid, response to cAMP, negative regulation of p38MAPK cascade, negative regulation of microglial cell activation, nervous system development and regulation of neuroinflammatory response. The pathways of homodirectional genes were significantly enriched in fluid shear stress and atherosclerosis and MAPK signaling pathway. Gene ontology-biological process (GO-BP) of opposite genes were enriched in neuron development, negative regulation of response to oxidative stress, neuron differentiation and regulation of cellular response to oxidative stress. The pathways of opposite genes were enriched in tyrosine metabolism, VEGF signaling pathway, regulation of lipolysis in adipocyte, adipocytokine signaling pathway and dopaminergic synapse.

There were 236 DEGs and 797 DTGs between the AD and AD_HFD groups, and 19 genes that changed at both levels. The combination of differences based on transcriptional and translational analysis revealed that *Ecm1, Reep4, and Cmtm3* were both up-regulation; *Gbp5*, *H1f3*, and *H1f4* were both down-regulated; *Sspo*, *Hoxb5*, and *Ccm2* were up-regulation in transcriptome and down-regulated in translatome; *Slc1a1*, *Glt8d2*, *Serinc2*, *Cd34*, *C1ra*, *Thbs4*, *Lct*, *Gm45208*, *Ltf*, and *Cnpy1* were down-regulated in transcriptome and up-regulated in translatome. The GO-BP of homodirectional genes had function at cellular process and positive regulation of biological process. *H1f3*, *H1f4* were closely associated with histone modification. The pathways of homodirectional genes were significantly enriched in nucleotide-binding oligomerization domain (NOD)-like receptor signaling pathway. The GO-BP of opposite genes had function at metabolic process, cellular process, biological regulation and developmental process. *Ccm2, Cd34, Thbs4, and Slc1a1* were closely associated with blood vessel development. Opposite genes were enriched in phagosome, galactose metabolism, carbohydrate digestion and absorption, synaptic vesicle cycle.

There were 203 DEGs and 1143 DTGs between the AD_HFD and H_H groups, and 18 genes that changed at both levels. The combination of differences based on transcriptional and translational analysis revealed that *Alms1, Lcmt2, Ryr3, and Ppp1r10* were both up-regulated; *Zfp968, Ccn1, Npas4, Fos, Dusp1, and Mpeg1* were both down-regulated; *C1ra, Mpp4, and Thbs4* were up-regulated in transcriptome and down-regulation in translatome; *Otof, Abl2, Glra1, Hoxb5*, and *Nrap* were down-regulated in transcriptome and up-regulated in translatome. The GO-BP of homodirectional genes were enriched in response to endogenous stimulus, learning, positive regulation of ceramide biosynthetic process, regulation of ceramide biosynthetic process, regulation of metabolic process, response to lipid and cognition. The pathways of homodirectional genes were significantly enriched in MAPK signaling pathway, Th1 and Th2 cell differentiation, IL-17 signaling pathway, TNF signaling pathway and dopaminergic synapse. The GO-BP of opposite genes were enriched in endothelial cell-cell adhesion, negative regulation of transmission of nerve impulse and behavior. The pathways of homodirectional genes were significantly enriched in ErbB signaling pathway and ECM-receptor interaction (Figure 2D).

Some of the genes were regulated differently in transcriptome and translatome. These outcomes suggested that regulation of translation had a relatively isolated role in regulating gene expression compared to regulation of transcription, and suggested sometimes translational regulation might completely reverse the effects of transcriptional regulation.

#### Analysis of differentially expressed genes and DTEGs

Using Ribo-seq and mRNA-seq data from the same sample, the translation efficiency (TE) of each gene was calculated. It exhibited a very weak correlation between TE and transcription abundance in four groups. 63 genes between the Nor and AD groups were significantly different at TE and transcription and had the opposite trends (opposite). The opposite genes were mainly enriched in the aspects of aging, neurotransmitter loading into synaptic vesicle, response to endogenous stimulus and dopamine metabolic process terms. The pathways were significantly enriched in cocaine addiction, amphetamine addiction, alcoholism, tyrosine metabolism, dopaminergic synapse and caffeine metabolism.

In total of 62 genes between the AD and AD_HFD groups were opposite. The genes were mainly enriched in neurotransmitter loading into synaptic vesicle, aminergic neurotransmitter loading into synaptic vesicle, response to nicotine, neurotransmitter transport, regulation of neurotransmitter levels terms in biological process ontology. The pathways were significantly enriched in neuroactive ligand-receptor interaction, dopaminergic synapse, synaptic vesicle cycle, alcoholism, and tyrosine metabolism.

*Zfp968* and *Ccn1* between the AD_HFD and H_H groups were significantly different at both levels and had the same tendency. 60 genes between AD_HFD and H_H groups were opposite. The genes were mainly enriched in skeletal system morphogenesis, embryonic skeletal system morphogenesis and neuropeptide signaling pathway terms. The pathways were significantly enriched in neuroactive ligand-receptor interaction, galactose metabolism, carbohydrate digestion and absorption.

### Differential expression analysis of no coding RNAs

**FIGURE 5:**
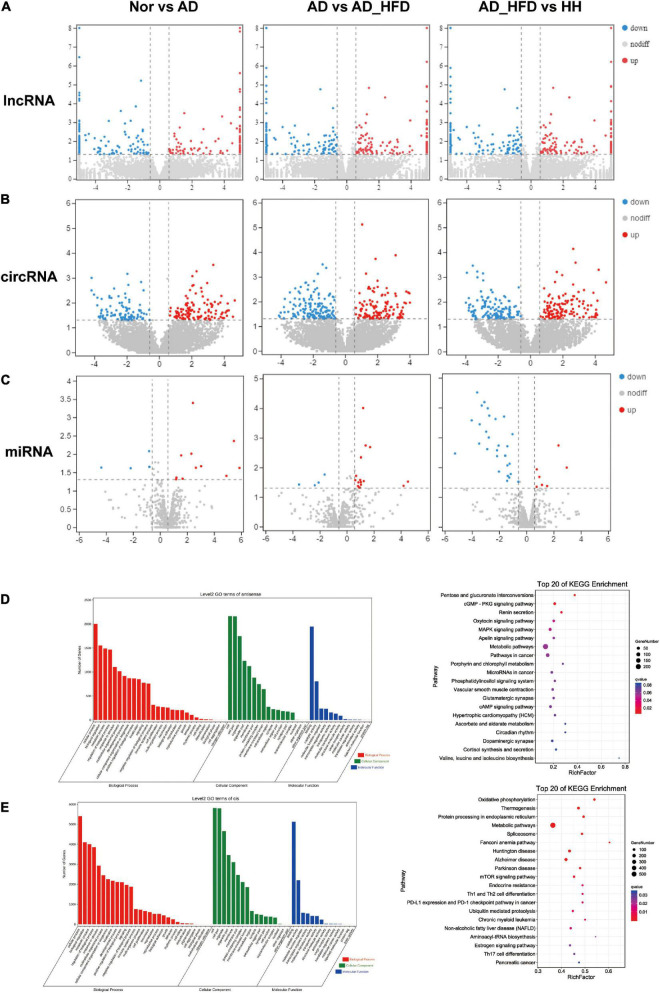
No coding RNAs sequence analysis. Differentially expressed genes heatmaps of lncRNA-seq **(A)**, circRNA-seq **(B)**, and miRNA-seq **(C)**. GO and KEGG enrichment analysis of antisense gene **(D)** and cis gene **(E)**.

The number of lncRNA transcripts reconstruction using StringTie was 7777. A total of 24717 circRNAs were identified in brain tissue samples, including 713 existing circRNAs and 24004 newly predicted circRNAs. A total of 1516 miRNAs were identified in mouse brain tissue samples. The length distribution obtained by miRNA sequencing of all samples was only one peak at 22bp. LncRNAs, circRNAs and miRNAs with *P* < 0.05 and | log_2_FC| ≥ 0.585 were screened as significant DEGs. According to the screening criteria, 114 or 137 were up- or down-regulated differentially expressed lncRNAs (dif-lncRNAs), 154 or 159 were up- or down-regulated differentially expressed circRNAs (dif-circRNAs), 11 or 5 were up- or down-regulated differentially expressed miRNAs (dif-miRNAs) in the AD group compared to the Nor group. There were 126 and 138 dif-lncRNAs, 193 and 202 dif-circRNAs, 17 and 4 dif-miRNAs up and down regulated in the AD_HFD group compared to the AD group. 150 and 129 dif-lncRNAs, 174 and 207 dif-circRNAs and 7 and 29 dif-miRNAs up and down regulated in the H_H group compared to the AD_HFD group (Figures 5A–C).

#### Long non-coding RNA analysis

Because of the complex origin of lncRNAs and the large variation in lncRNAs produced by different transcripts of the same gene, lncRNAs would be analyzed by transcript. The coding ability of new transcripts was predicted by CPC2 and CNCI software (Supplementary Figure 5A). The intersection of these non-coding potential transcripts was taken as a reliable predictor of the outcome. 734 transcripts with no coding ability were predicted. We performed *de novo* lncRNA prediction (Supplementary Figure 5C).

#### Long non-coding RNA-mRNA association analysis

Long non-coding RNAs were involved in the regulation of many post-transcriptional processes, and were similar to small RNAs such as miRNAs and snoRNAs. These regulations were often associated with complementary pairing of bases. A fraction of antisense lncRNAs might regulate gene silencing, transcription and mRNA stability due to binding to mRNAs of the righteous strand. To reveal the interactions between antisense lncRNAs and mRNAs, we used RNAplex (47) to predict complementary binding between antisense lncRNAs and mRNAs.

We predicted antisense effects to obtain 3718 lncRNA-mRNA target gene pairs and cis effects to obtain 14398 lncRNA-mRNA target gene pairs. The pathways that were significantly enriched in KEGG of antisense effects were pentose and glucuronate interconversions, MAPK signaling pathway, apelin signaling pathway and metabolic pathways (Figure 5D). The pathways that were significantly enriched in KEGG of cis effects were oxidative phosphorylation, metabolic pathways, Alzheimer’s disease, Parkinson’s disease and mTOR signaling pathway. Cis effects of lncRNA-mRNA might be more involved in this AD experiment process (Figure 5E).

#### Circular RNA analysis

**FIGURE 6:**
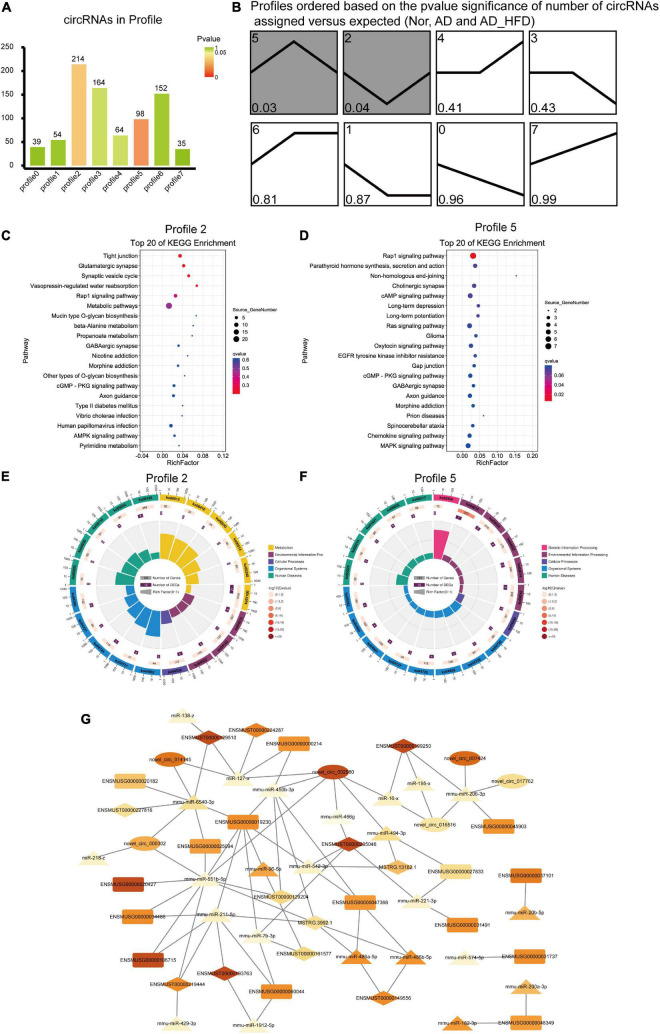
Trend analysis of circRNAs and ceRNA network. **(A)** The number of circRNAs in profile. Green represents *P*-value which is closed to 1 and red represents the *P*-value is closed to 0. **(B)** Trends analysis of circRNAs among the Nor, AD and AD_HFD groups. Pathways enriched by genes regulated by differentially expressed circRNAs in profile 2 **(C)** and profile 5 **(D)**. The enrichment circle diagrams of GO enrichment analysis in profile 2 **(E)** and profile 5 **(F)**. The first circle: enrichment of the first 20 GO term. Different colors represent different ontologies; the second circle: the number and *Q*-value of the GO term in each classification gene background. The more the number of gene background in each classification, the longer the bar, and the smaller the *Q*-value, the redder the color. The third circle: the number of genes in each category of the GO term; fourth circle: RichFactor value of each GO term. **(G)** The Competing Endogenous RNA Network. Rectangle indicates mRNA, ellipse indicates circRNA, and prism indicates lncRNA, triangle indicates miRNA. The larger the FC value, the darker the color.

Trend analysis was used to observe the tendency in circRNA variation among the Nor, AD and AD_HFD groups. There were 820 dif-circRNAs in three groups, with significance in profile 2 and profile 5 (Figures 6A,B). These genes had a change trend of callback, suggesting that such circRNA source genes might be involved in the influence process of the HFD intervention on AD. KEGG enrichment analysis revealed that the pathways significantly enriched in genes of profile 2 were glutamatergic synapse, synaptic vesicle recycle, Rap1 signaling pathway, alanine metabolism, propanoate metabolism and GABAergic synapses (Figure 6C). Pathways significantly enriched in profile 5 were Rap1 signaling pathway, cholinergic synapse, cAMP signaling pathway, long-term depression, long-term potentiation and RAS signaling pathway (Figure 6D). The enrichment circle diagrams of GO enrichment analysis were shown in Figures 6E,F.

#### Competing endogenous RNA analysis

Screening of mRNAs, lncRNAs, miRNAs and circRNAs in the AD and AD_HFD groups yielded 164, 225, 50, and 309 differential genes, respectively. The miRNA-target gene pairs were predicted and screened for target gene pairs with Spearman’s correlation coefficient less than or equal to 0.5, combined with ceRNA pairs with the positive expression correlation (Pearson’s correlation coefficient more than 0.7) to obtain potential ceRNA pairs, and then screened for ceRNA pairs with *P*-value less than 0.05 as the final ceRNA pairs using hypergeometric distribution test.

Mmu-miR-551b-5p was competitively integrated by *Igfbp3* (ENSMUSG00000020427), *Slc18a2* (ENSMUSG000000 25094), *Tmem265* (ENSMUSG00000106715), *Gbx2* (ENSMU SG00000034486), *Lhx9* (ENSMUSG00000019230), ENSM UST00000219444, and MSTRG.3992.1. Mmu-miR-211-5p was competitively integrated by *Lhx9* (ENSMUSG00000019230), *Tmem26* (ENSMUSG00000060044), *Tmem265* (ENSMUSG 00000106715), *Gbx2* (ENSMUSG00000034486), ENSMUST000 00219444, and MSTRG.3992.1. Mmu-miR-6540-3p was competitively integrated by *Slc18a2* (ENSMUSG00000025094), *Lhx9* (ENSMUSG00000019230), *Ddc* (ENSMUSG00000020 182), novel_circ_014145, novel_circ_000302, ENSMUST0000 0129510, ENSMUST00000227816. Mmu-miR-221-3p was competitively integrated by *Chrna6* (ENSMUSG00000031491), *Shox2* (ENSMUSG00000027833). *Pou4f1* (ENSMUSG0 0000048349) was regulated by mmu-miR-200a-3p, mmu-miR-182-3p. Mmu-miR-450b-3p was competitively integrated by *Th* (ENSMUSG00000000214), *Lhx9* (ENSMUSG00000019230), *Abhd17b* (ENSMUSG00000047368), novel_circ_002080, ENSMUST00000129204, MSTRG.3992.1 (Figure 6G).

## Discussion

Among all the relationship networks of ceRNAs, mmu-miR-450b-3p and mmu-miR-6540-3p regulated the expression of *Th* and *Ddc*, respectively. Both of them were closely related to the regulation of catecholamine neurotransmitters. A variety of lncRNAs and circRNAs were also involved in the regulation of their gene expression, and the specific mechanisms needed to be further validated and discovered. Insulin-like growth factor binding protein (IGFBP) was a family of proteins with high affinity for insulin-like growth factor (IGF). IGF-1 and IGFBP-3 were associated with oxidative stress and longevity (48). IGF-1 was thought to be a typical neuronal pro-survival factor in various brain injuries, promoting the clearance of Aβ and suppressing inflammatory responses. It could also affect cognitive performance by regulating synaptic plasticity, synaptic density and neurotransmission (49).

In addition to regulating IGF activity, IGFBP3 could also independently regulate cell growth and survival. IGFBP3 could bind and regulate retinoid X receptor α, upregulate pro-apoptotic signaling pathways such as TNFα and TGFβ (50). Current experimental studies and epidemiological findings on its relevance to AD were controversial, with some studies suggesting that higher serum total IGF-I levels and higher total IGF-I/IGFBP-3 ratios were associated with less cognitive decline (51). Low serum levels of IGF-1 and IGFBP-3 in male individuals were associated with AD (52). IGFBP-3 inhibited Aβ_42_-induced apoptosis and long-term exposure to Aβ_42_ could induce IGFBP-3 hypermethylation (53). In contrast, study suggested that Aβ_42_ upregulate the expression of IGFBP3 (54), and the increased IGFBP3 expression was seen in senile plaques and neurofibrillary tangles (55). Aβ could activate calcium-regulated phosphatases in astrocytes, causing the release of IGFBP3, which in turn induced tau protein phosphorylation (56).

In this study, *Igfbp3* expression was reduced in the AD group, and its expression was significantly upregulated by HFD intervention, and its gene expression level was further increased by HLJDD administration. Further studies on the IGFBP3 were still needed to clarify its effect on the course of AD. LncRNA *Rmst-208* (ENSMUST00000219444), MSTRG.3992.1 in the ceRNA network were competed with *Igfbp3* to bind mmu-miR-551b-5p. In addition, *Pou4f1* in the competition network inhibited neuronal apoptosis, *Slc18a2* negatively regulated neurotransmitter transport, *Shox2* and *Irx5* were associated with neurodevelopment, and the transmembrane protein TMEM also played an important role in human immune-related diseases as well as tumor development (57, 58). *Slc18a* was associated with the regulation of neurotransmitter transport, which was regulated by mmu-miR-6540-3p and miR-551b-5p. ceRNA analysis revealed that *Lhx9*, lncRNA *Acbd5* competed with *Slc18a2* to bind mmu-miR-6540-3p. *Tmem265*, *Gbx2*, *Lhx9*, lncRNA *Rmst-208* and MSTRG.3992.1 competes with I*gfbp3* to bind mmu- miR-551b-5p. The prognostic value and underlying mechanisms of the miRNAs, lncRNAs and circRNAs that we identified needed to be further studied.

Interestingly, we also had a group of normal mice giving the HFD intervention in animal housing. However, this group was not performed the transcriptional and translational experiments. Compared to the normal diet, the HFD intervention resulted in a reduction in the number of platform penetrations, the percentage of platform quadrant distances and times in the normal mice. Whereas in the AD mice, on the contrary, the HFD intervention tended to ameliorate the cognitive impairment in the transgenic mice. This result meant that HFD had different effects on the animal. In the mRNA trend analysis and GSEA pathway enrichment results, most of the pathways enriched by DEGs in the AD group were related to the metabolism of neurotransmitter-like substances. Our laboratory examined the concentration of amino acids and neurotransmitters in mice brain tissue and found significant changes in acetylcholine, GABA, glutamine, phenylalanine, lysine, arginine, proline and alanine in the AD and AD_HFD groups (10). The result indicated the DEGs were involved in the metabolism of amino acids and neurotransmitters in the brain tissue of AD mice. We confirmed the HFD modulated brain tissue levels of serotonin, choline, tryptophan, GABA, glycine, phenylalanine, methionine, hypoxanthine and homovanillic acid in AD mice. In the present study, we also found that HFD could modulate the gene changes in profile 2 (transcriptome), and affect the metabolism of neurotransmitters in the brain tissue. In addition, IGFBP was associated with apoptosis and tau protein phosphorylation. The increased transcription of *Igfbp3* in the AD_HFD and H_H groups might be related to its cognitive impairment.

PCSK9 was found to promote LDL degradation. The upregulation of *Pcsk9* expression in the AD_HFD group might be closely related to the increase of cholesterol in their brain tissues, while HLJDD significantly downregulated the expression of *Pcsk9*. SLC10A4 was a family of sodium bile acid cotransport proteins that were activated by proteases to participate in the transport of bile acids in brain tissue. The expression of *Slc10a4* was significantly decreased in AD group and significantly increased in AD_HFD group. HLJDD could significantly reduce the expression of *Slc10a4*. CYP27A1 regulated the synthesis of primary bile acids in the alternative pathway. The results of the previous experiments on serum bile acids in mice also showed that HFD increased the level of CDCA produced by the alternative pathway in mice. This result once again confirmed that HFD intervention could cause the transformation in the bile acid synthesis pathway in AD mice.

During the imposed remodeling of gene expression, transcription level alterations of certain mRNA didn’t closely correlate with those of the encoded proteins, which could partially depend on the differential recruitment of mRNAs to translate ribosomes. Translatome could provide vital information for the translational regulation, to study the process of protein production from mRNA translation. The translational response helped to establish complex genetic regulation that couldn’t be achieved by controlling transcription alone. This suggested that the roles of translational and transcriptional regulation were relatively independent. A large amount of data still needed to be mined in depth to discover more valuable regulatory networks, which would provide a basis and direction for later studies on AD and HFD intervention mechanisms, thus providing a more comprehensive understanding of the occurrence and development of AD disease. In summary, our analysis revealed distinct and related roles for translational and transcriptional regulation in HFD on AD mice, highlighting a critical role of translational regulation on AD.

## Data availability statement

The datasets presented in this study can be found in online repositories. The names of the repository/repositories and accession number(s) can be found here: http://bigd.big.ac.cn/gsa/, CRA007307.

## Ethics statement

The animal study was reviewed and approved by Institutional Animal Care and Use Committee of the Beijing Animal Science Co., Ltd., and the animal ethics approval number was IACUC-2018100605.

## Author contributions

WG, JZ, XF, LW, and XG performed the experiments. WG analyzed the data and wrote the original manuscript. YZ and HZ revised the manuscript. NS and HW contributed to the work. BB and HZ conceived and designed the experiments. All authors reviewed the manuscript and approved the submitted version.
