# Supplementary material for: A multi-network comparative analysis of whole-transcriptome and translatome reveals the effect of high-fat diet on APP/PS1 mice and the intervention with Chinese medicine
Source: Front Nutr. 2022 Oct 24;9:974333. doi: 10.3389/fnut.2022.974333 (PMC9638104; doi:10.3389/fnut.2022.974333)
Supplement: Supplementary file 1 [file Data_Sheet_1.docx]

***Supplementary Material***

**1 Supplementary Data**

**RNA-Seq**

**mRNA, lncRNA and circRNA Library construction and sequencing**

Total RNA was extracted using Trizol reagent kit (Invitrogen, Carlsbad, CA, USA) according to the manufacturer’s protocol. After total RNA was extracted, rRNA was removed by using VAHTS Total RNA-seq (H/M/R) Library Prep Kit for Illumina (Vazyme Biotech Co., Ltd, Nanjing, China) to retain other types of RNA, including mRNAs and ncRNAs. The enriched mRNAs and ncRNAs were fragmented into short fragments in fragmentation buffer (MgCl_2_) integrated into VAHTS Total RNA-seq (H/M/R) Library Prep Kit, then the RNA fragments were reverse-transcribed into cDNA with random primers. Second-strand cDNA was synthesized with DNA polymerase I, RNase H, dNTPs (dUTP instead of dTTP) and buffer. Next, the cDNA fragments were purified with a QiaQuick PCR extraction kit, end-repaired, underwent poly(A) addition, and ligated to Illumina sequencing adapters. Then, uracil-N-glycosylase (UNG) was used to digest the second-strand cDNA. The digested products were size-selected via agarose gel electrophoresis, PCR-amplified, and sequenced using an Illumina HiSeqTM 4000.

**smallRNA Library construction and sequencing**

After total RNA was extracted by Trizol reagent kit (Invitrogen, Carlsbad, CA, USA), the RNA molecules in a size range of 18–30 nt were enriched by polyacrylamide gel electrophoresis (PAGE). Then the 3’ adapters were added and the 36-48 nt RNAs were enriched. The 5’ adapters were then ligated to the RNAs as well. The ligation products were reverse transcribed by PCR amplification and the 140-160 bp size PCR products were enriched to generate a cDNA library and sequenced using Illumina HiSeq Xten.

**Bioinformatics analysis and identification of target mRNA and lncRNA**

Short reads alignment tool Bowtie2 (version 2.2.8) was used for mapping reads to ribosome RNA (rRNA) database. The rRNA mapped reads were then removed. The remaining reads were further used in assembly and analysis of transcriptome. An index of the reference genome was built, and paired-end clean reads were mapped to the reference genome using HISAT2 (version 2.1.0) with “rna-strandness RF” and other parameters set as a default. The reconstruction of transcripts was carried out with software Stringtie (version 1.3.4). To identify the new transcripts, all of the reconstructed transcripts were aligned to reference genome and were divided into twelve categories by using Cuffcompare. Transcripts with one of the classcodes “u,i,j,x,c,e,o” were defined as novel transcripts. We used the following parameters to identify reliable novel genes: the length of transcript was longer than 200 bp and the exon number was more than 2 (plants more than 1). Novel transcripts were then aligned to the Nr, KEGG, and GO database to obtain protein functional annotation. Two softwares, CNCI (version 2) and CPC (version 0.9-r2) (http://cpc.cbi.pku.edu.cn/), were used to assess the protein-coding potential of novel transcripts by default parameters. The intersection of both non protein-coding potential results were chosen as long non-coding RNAs. lncRNAs were classified into five classes according to their location relative to protein-coding genes: intergenic lncRNAs, bidirectional lncRNAs, intronic lncRNAs, antisense lncRNAs, and sense overlapping lncRNAs. Different types of lncRNAs may have different biological functions. For each transcription region, a FPKM (fragment per kilobase of transcript per million mapped reads) value was calculated to quantify its expression abundance and variations, using RSEM software. Principal component analysis (PCA) was performed with R package gmodels (http://www.rproject.org/) in this experience. The differentially expressed transcripts of coding RNAs and lncRNAs were analyzed respectively. RNAs and lncRNAs differential expression analysis was performed by DESeq2 software between two different groups (and by edgeR between two samples). The genes/transcripts with the parameter of false discovery rate (FDR) below 0.05 and absolute |log_2_FC| ≥ 0.585 were considered differentially expressed genes/transcripts. Differentially expressed coding RNAs were then subjected to enrichment analysis of GO functions and KEGG pathways. We performed gene set enrichment analysis using software GSEA and MSigDB. Enrichment scores and p value was calculated in default parameters. Protein-Protein interaction network was identified using String v10. To better annotate lncRNA in evolution level, the software Infernal (version 1.1.2) was used in sequence alignment. lncRNAs were classified by secondary structures and sequence conservation. To find potential miRNA precursors, lncRNAs were aligned to miRbase (version 21). Those with identity more than 90% were selected. In addition, the software miRPara (version 6.3), which is based on SVM method was also used to predict miRNA precursors. After mapping reads to reference genome, the Cufflink was used in reconstruction of transcripts which may extend the 5' untranslated region (5’ UTR) or 3’ UTR of gene as optimizing the gene structure. The GATK (version 3.4-46) was used for calling variants of transcripts, and ANNOVAR was used for SNP/InDel annotation. The function, genome site and type of variation of SNPs were also analyzed. rMATS (version 4.0.1) (http://rnaseq-mats.sourceforge.net/index.html) was used to identify alternative splicing events and analyze differential alternative splicing events between samples. We identified AS events with a false discovery rate (FDR) <0.05 in a comparison as significant AS events. In order to reveal the interaction between antisense lncRNA and mRNA, the software RNAplex (version 0.2) (http://www.tbi.univie.ac.at/RNA/RNAplex.1.html) was used to predict the complementary correlation of antisense lncRNA and mRNA.

**Bioinformatics analysis and identification of target circRNAs**

Short reads alignment tool Bowtie2 (version 2.2.8) was used for mapping reads to ribosome RNA (rRNA) database. The rRNA mapped reads would be removed. The remaining reads were further used in alignment and analysis. The rRNA removed reads of each sample were then mapped to reference genome by TopHat2 (version 2.1.1), respectively. After aligned with reference genome, the reads that could be mapped to the genomes were discarded, and the unmapped reads were then collected for circRNA identification. 20mers from both ends of the unmapped reads were extracted and aligned to the reference genome to find unique anchor positions within splice site. Anchor reads that aligned in the reversed orientation (head-to tail) indicated circRNA splicing and then were subjected to find_circ (version 1) to identify circRNAs. The anchor alignments were then extended such that the complete read aligns and the breakpoints were flanked by GU/AG splice sites. A candidate circRNA was called if it was supported by at least two unique back spliced reads at least in one sample. The identified circRNAs were subjected to statistical analysis of type, chromosome distribution and length distribution. Source gene is the origin gene of circRNA. The functional enrichment analysis of source genes was performed to study the main functions of these source genes of circRNAs. To quantify circRNAs, back-spliced junction reads were scaled to RPM (Reads Per Million mapped reads), and the formula is RPM=10^6^C/N. In this formula, C is the number of back-spliced junction reads that uniquely aligned to a circRNA. N is the total number of back-spliced junction reads. The RPM method is able to eliminate the influence of different sequencing data amount on the calculation of circRNA expression. Therefore, the calculated expression can be directly used for comparing the differential expression among samples. To identify differentially expressed circRNAs across samples or groups, the edgeR package (version 3.12.1) (http://www.r-project.org/) was used. We identified circRNAs with a |log_2_FC| ≥ 0.585 and a P value < 0.05 in a comparison between samples or groups as significant differentially expressed circRNAs. circRNAs were blasted against the circBase for annotation. Those cannot be annotated were defined as novel circRNAs. For circRNAs that have been annotated in circBase, the target relationship with miRNAs can be predicted by StarBase (version 2.0). For novel circRNAs, three softwares: Mireap, Miranda (version 3.3a) and TargetScan (version 7.0) were used to predict targets. For prediction of mRNAs interacting with circRNAs and miRNAs, miRTarBase (version 6.1) was used to predict mRNAs targeted by miRNAs sponge. The resulting correlation of circRNAs-miRNAs-mRNAs could be visualized by Cytoscape.

The cORF pipeline script was used to search circRNA with ORF spanning junction site of circRNA. Each circRNA sequence, excluding intronic sequences, was multiplied four times, and the longest ORF spanning the circRNA junction was selected for each one of the three frames (minimum cORF length threshold was 20aa). The IRESfinder was used to identify the IRES sequence of circRNA. Each sequence of circRNA was evaluated by sliding window approach with window size of 174 nt and step size of 50 nt. The region with the highest score was the IRES sequence of circRNA. The junction reads with Ribo-seq data were searched following the method from Salzman’s study. In briefly, a custom database of exon-exon junction sequence was constructed for all exon-pairs related the junction site of circRNA. After aligned with reference genome by bowtie2, the reads mapping to genome which derived from linear RNA were discarded. The remained unmapped reads which potentially harboring junction reads of circRNA were collected for further analysis. Then, the unmapped reads were aligned to custom database of exon-exon junction sequence to find the reads from junction site of circRNA. We did not allow any mismatches and required a minimum read-junction overlap of 9 nt on either side of the junction. The number of junction reads which indicated translation signal was calculated for each circRNA. We performed the functional enrichment analysis of source genes set of circRNAs with junction reads.

**Construction of ceRNA network**

To identify differentially expressed transcripts across samples or groups, the edgeR package (http://www.bioconductor.org/packages/release/bioc/html/edgeR.html) was used. We identified mRNA and lncRNA with a |log_2_FC| ≥ 0.585 and a false discovery rate (FDR) < 0.05 in a comparison as significant differentially expressed genes (DEGs), and miRNA with a |log_2_FC| ≥ 0.585 and p < 0.05. Three softwares, mireap, miRanda and TargetScan, were used to predict miRNA targets. miRNA sequences and family information were obtained from TargetScan website (<http://www.targetscan.org/>). We combined mRNA, miRNA, lncRNA and circRNA data from whole transcriptomic sequencing to construct the ceRNA regulatory networks. In order to ensure the reliability, reliable RNA molecules could be analyzed, and the newly predicted mRNA and the new miRNA would not be included in the association analysis of ceRNA because of high false positives. We integrated the co-expression relationship of lncRNA, mRNA and the regulation relationship of dif-miRNA-dif-mRNA and dif-miRNA-dif-lncRNA. The association analysis of ceRNAs was selected from pre-existing miRNAs. Screening for ceRNAs with potential interactions would be filtered by three aspects, including: targeting relationships between miRNAs and candidate ceRNAs and negative correlations in expression; positive correlations in expression between candidate ceRNAs; and the degree of enrichment of candidate ceRNAs binding the same miRNA. The lncRNA-miRNA-mRNA complex network was constructed by Cytoscape. Similarly, we integrated the co-expression relationships of circRNA-mRNA and the regulatory relationships of dif-miRNA-dif-mRNA and dif-miRNA-dif-circRNA. We were more concerned with the positive correlated expression of lncRNA-mRNA, so we focused on miRNAs that could regulate both circRNA and mRNA, and further obtained positive correlated co-expression relationships between mRNAs and circRNAs regulated by these miRNAs. The circRNA-miRNA-mRNA network was constructed using Cytoscape to integrate the two complex networks of circRNA-miRNA-mRNA and lncRNA-miRNA-mRNA, and we focused on screening miRNAs that could regulate circRNA, lncRNA and mRNA. furthermore, we obtained the positive correlation co-expression relationships of mRNA, lncRNA and circRNA regulated by these miRNAs were further obtained. Cytoscape was applied to establish the ceRNA network.

**Ribo-Seq**

Tissues were immediately frozen in liquid nitrogen and ground to power in liquid nitrogen with a mortar and pestle, then dissolved in 400 µL of lysis buffer. The resuspended extracts in lysis buffer were transferred to new microtubes, pipetted several times and incubated on ice for 10 min. Then cells were triturated ten times through a 26-G needle. The lysate was centrifuged at 20000 g for 10 min at 4℃. The supernatant was collected. To prepare ribosome footprints (RFs), 7.5 µL of RNase I and 5 µL of DNase I were added to 300 µL of lysate to incubate for 45 min at room temperature with gentle mixing on a Nutator mixer. Nuclease digestion was stopped by adding 10 µL of SUPERase·In RNase inhibitor. Size exclusion columns (illustra MicroSpin S-400 HR Columns; GE Healthcare; catalog no. 27-5140-01) were equilibrated with 3 mL of polysome buffer by gravity flow and centrifuged at 600 g for 4 min at room temperature. 100 μL of digested RFs were added to the column and centrifuged at 600 g for 2 min. Next, 10 μL 10% (wt/vol) SDS was added to the elution, and RFs with a size greater than 17 nt was isolated according to the RNA Clean and Concentrator-25 kit (Zymo Research; R1017). rRNA was removed using DNA probes complementary to rRNA sequences. Then RNase H and DNase I was used to digest the probes. RFs were purified using magnet beads (Vazyme). After obtaining ribosome footprints above, Ribo-seq libraries were constructed using NEBNext® Multiple Small RNA Library Prep Set for Illumina® (catalog no.E7300S, E7300L). Briefly, adapters were added to both ends of RFs, followed by reverse transcription and PCR amplification. The 140-160 bp size PCR products were enriched to generate a cDNA library and sequenced using Illumina HiSeqTM 2500 by Gene Denovo Biotechnology Co. (Guangzhou, China).

**Ribo-seq data analysis**

Raw reads containing over 50% of low quality bases or over 10% of N bases were removed. Adapter sequences were trimmed. Reads with length between 10-50 bp were retained for subsequent analysis. Short reads alignment tool Bowtie2 was used for mapping reads to ribosome RNA (rRNA) database. The rRNA mapped reads will be removed. The remaining reads were further used in downstream analysis. The rRNA removed reads of each sample were mapped to reference genome by Bowtie2 allowing no mismatches. RFs were assigned to different genomic features (5’UTR, CDS, 3’UTR and others) based on the position of the 5’ end of the alignment. To monitor sequencing reliability, RFs density at different codon positions was calculated. Reads number in the open reading frame of coding genes was calculated by software RiboTaper, and the gene expression level was normalized by using FPKM method. To evaluate the reliability of experimental results, the correlation coefficient between two replicas was calculated. Meanwhile, Principal component analysis (PCA) was performed with R package gmodels (http://www.r-project.org/) to reveal the relationship of the samples. To identify differentially translated genes across sample groups, the edgeR package (http://www.rproject.org/) was used. Genes with a |log_2_FC| ≥ 0.585 and a false discovery rate (FDR) <0.05 in a comparison were considered as significant DTGs. DTGs were then subjected to enrichment analysis of GO functions and KEGG pathways.

**Combination analysis of translatome and transcriptome**

According to the expression changes (|log_2_FC of FPKM| ≥ 0.585 and P < 0.05) at transcriptome and translatome, genes were classified into five groups: Transcription (significantly different only at transcriptional level); Translation (significantly different only at translational level); Homodirection (significantly different at both levels and have the same trends); Opposite (significantly different at both levels and have the opposite trends); Unchanged (not significantly different at both levels). Genes of the above five groups were subjected to enrichment analysis of GO functions and KEGG pathways respectively. Translational efficiency (TE) is the ratio of translating mRNAs to total mRNAs of a gene. The formula is shown as follows: TE = (FPKM in Ribo-seq) / (FPKM in RNA-seq). TE of genes were calculated and compared between samples and groups. To analysis the relationship between expression level and translational efficiency of genes, gene expression level and translational efficiency were plotted and pearson correlation coefficient was calculated. Diff-Ribo was used to identify differential TE genes across sample groups, and genes with a |log_2_FC| ≥ 0.585 and a false discovery rate (FDR) <0.05 in a comparison were considered as significant DTGs. DTGs were then subjected to enrichment analysis of GO functions and KEGG pathways. According to the expression changes at transcriptional level and the translational efficiency changes, genes were classified into five groups: Transcription (significantly different only at transcriptional level); TE (significantly different only at translational efficiency); Homodirection (significantly different at both dimensionalities and have the same trends); Opposite (significantly different at both dimensionalities and have the opposite trends); Unchanged (not significantly different at both dimensionalities). Genes of the above five groups were subjected to enrichment analysis of GO functions and KEGG pathways respectively. Gene differential expression analysis was performed using DESeq2 software. The reads count was normalized and the probability of hypothesis testing was calculated according to the model.

**2 Supplementary Figures and Tables**

**Supplementary Table 1**. Nucleotide sequences of gene-specific primers used for quantitative real-time PCR

| Gene name | Sequences of forward and reverse primers (5’ to 3’) | | Tm | CG% |
| --- | --- | --- | --- | --- |
| M-β-tubulin | sense | GAGCCAAGTTCTGGGAGGTG | 59.3 | 60 |
|  | antisense | CAGGCTGAAAGAGGACTCCC | 58.6 | 60 |
| M-TNF-α | sense | TCCCCAAAGGGATGAGAAGTT | 60.1 | 47.6 |
|  | antisense | GAGGAGGTTGACTTTCTCCTGG | 59.5 | 54.5 |
| M-IL-1β | sense | GGGCCTCAAAGGAAAGAATCT | 59.3 | 47.6 |
|  | antisense | GAGGTGCTGATGTACCAGTTGG | 59.5 | 54.5 |
| M-IL-6 | sense | CTTCTTGGGACTGATGCTGGT | 59.1 | 52.4 |
|  | antisense | CACAACTCTTTTCTCATTTCCACG | 59.1 | 52.4 |
| M-IL12A | sense | GCATGTGTCAATCACGCTACC | 58.7 | 52.4 |
|  | antisense | ATGTCATCTGTGGTCTTCAGCAG | 59.2 | 47.8 |
| M-IL12B | sense | GGACATCATCAAACCAGACCC | 58.8 | 52.4 |
|  | antisense | ACGCACCTTTCTGGTTACACC | 59.2 | 52.4 |
| M-IFN-γ | sense | CTCAAGTGGCATAGATGTGGAAG | 59.2 | 47.8 |
|  | antisense | GACCTCAAACTTGGCAATACTCA | 58.9 | 43.5 |
| M-MCP-1 | sense | CCCTACTATTCCTGATGGCACT | 59.02 | 50 |
|  | antisense | CTATGAGAAACCCACCACATCTG | 58.81 | 47.83 |


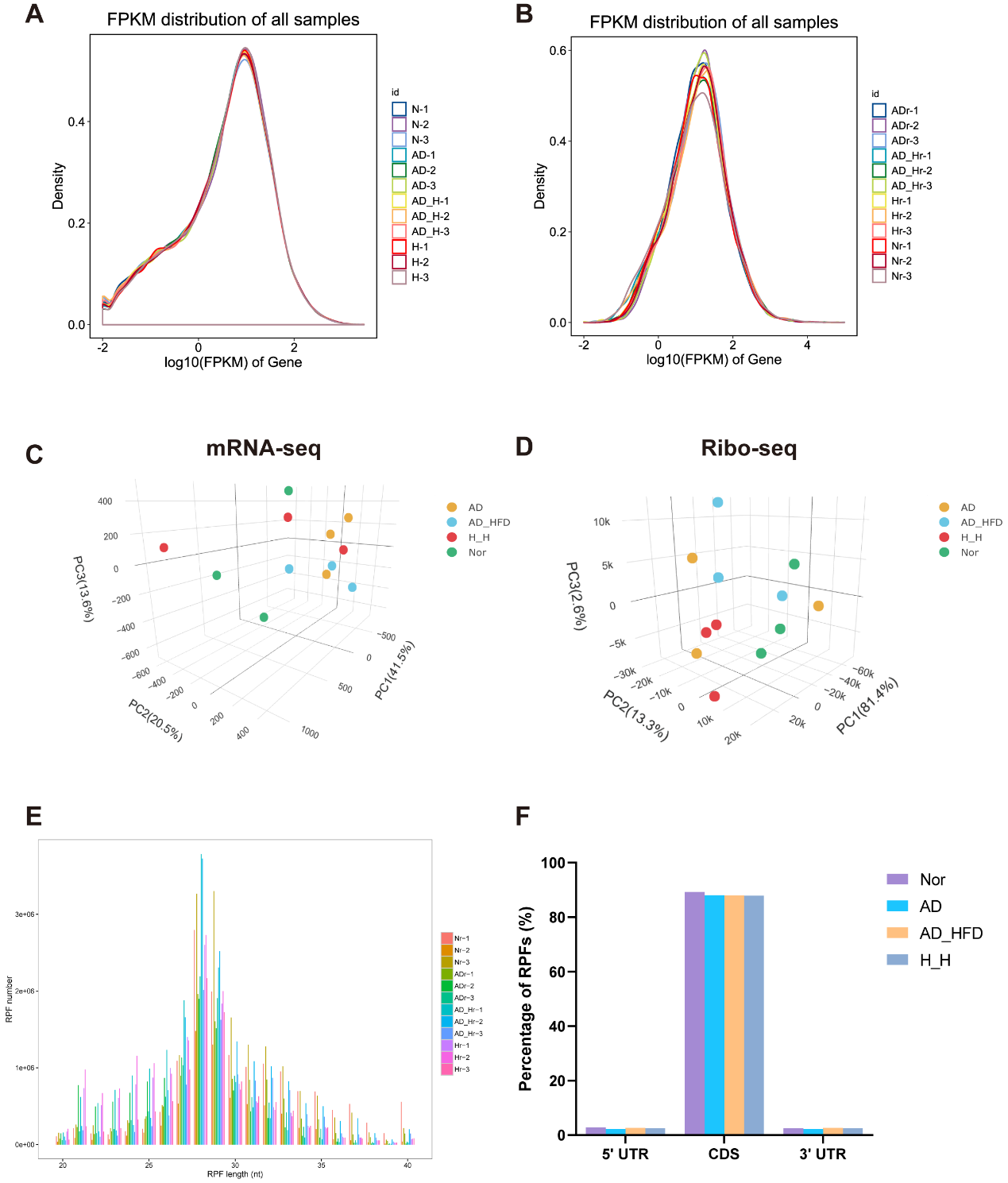


**Supplementary Figure 1** Overview of transcriptome and translatome.

Distribution of the abundance of each sample in transcriptome (A) and translatome (B). Principal-component analysis in transcriptome (C) and translatome (D) of each sample. (E) Length distribution of RPFs. (F) The percentage of RPFs located in the CDS, 5′UTR, and 3′UTR.


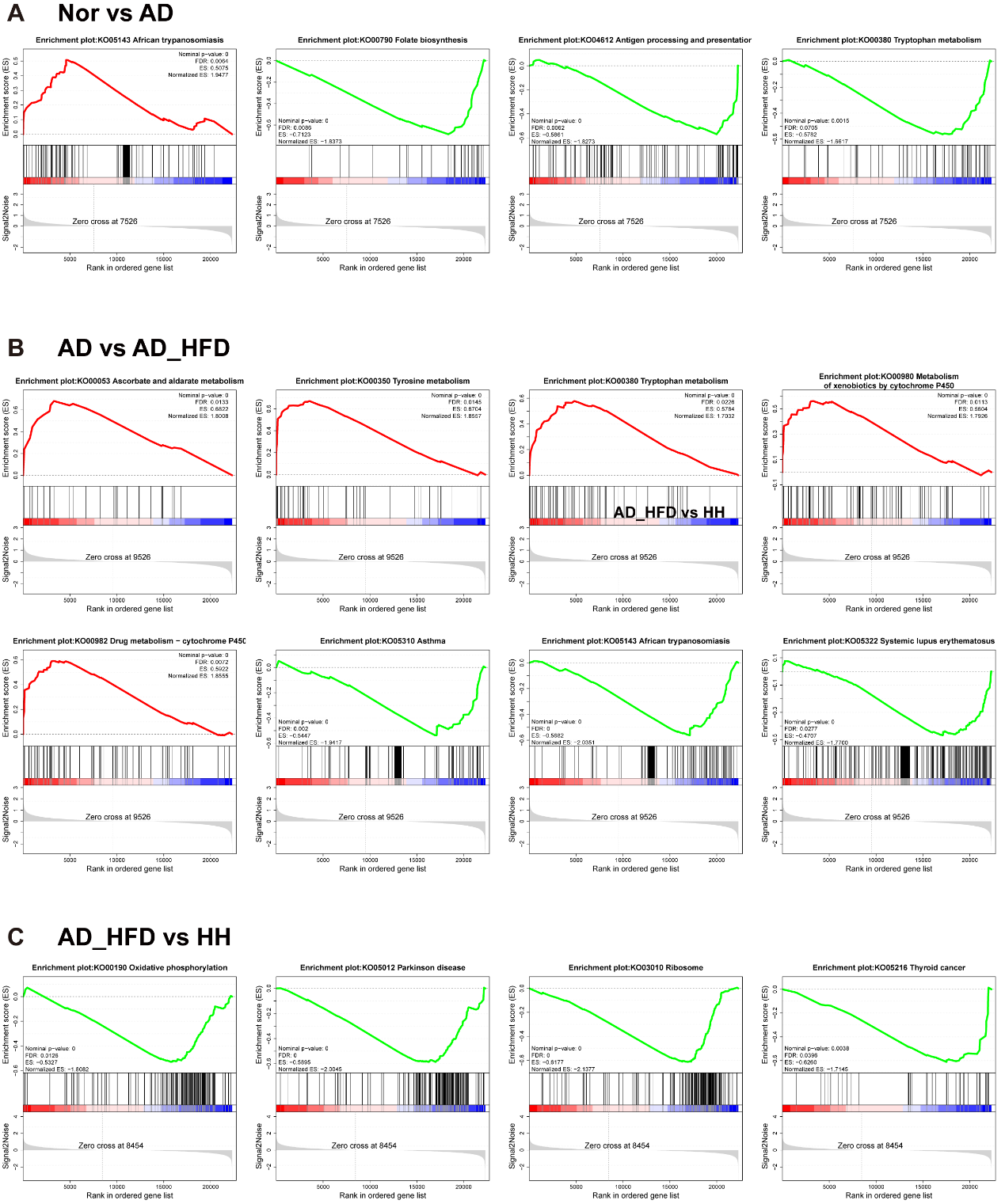


**Supplementary Figure 2** Some of GSEA in transcriptome. Pathways with positive enrichment scores are upregulated, while pathways with negative enrichment scores are downregulated.


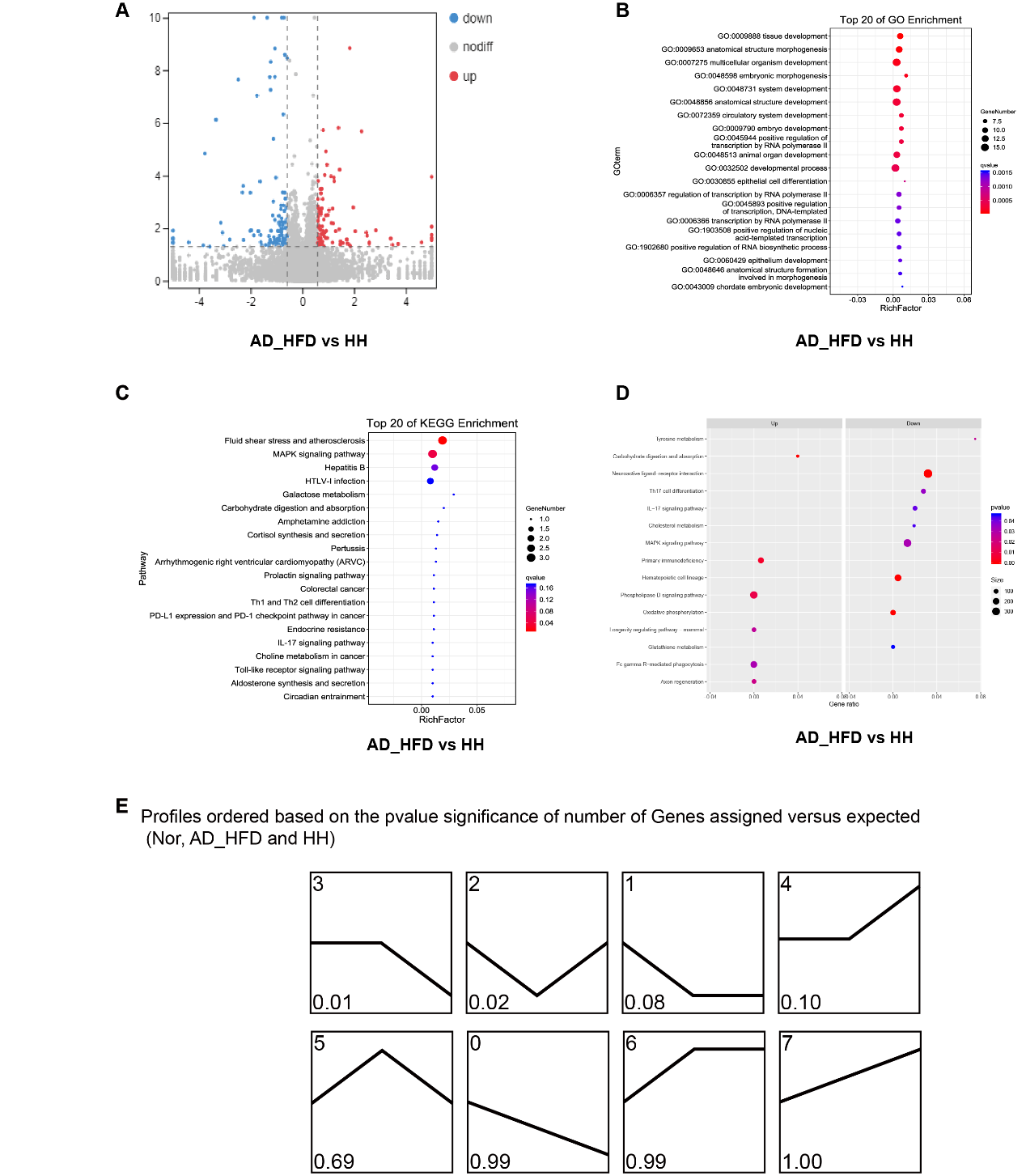


**Supplementary Figure 3** Transcriptome analysis in AD mice combined with HLJDD and HFD

(A) Volcano plots of differentially expressed mRNA between the AD_HFD and H_H groups in transcriptome. (B) GO enrichment analysis of the transcriptome between the AD_HFD and H_H groups. (C) KEGG pathway analysis of the transcriptome between the AD_HFD and H_H groups. (D) GSEA of the transcriptome between the AD_HFD and H_H groups. (E) Trends in genetic variation among the AD, AD_HFD and H_H groups.


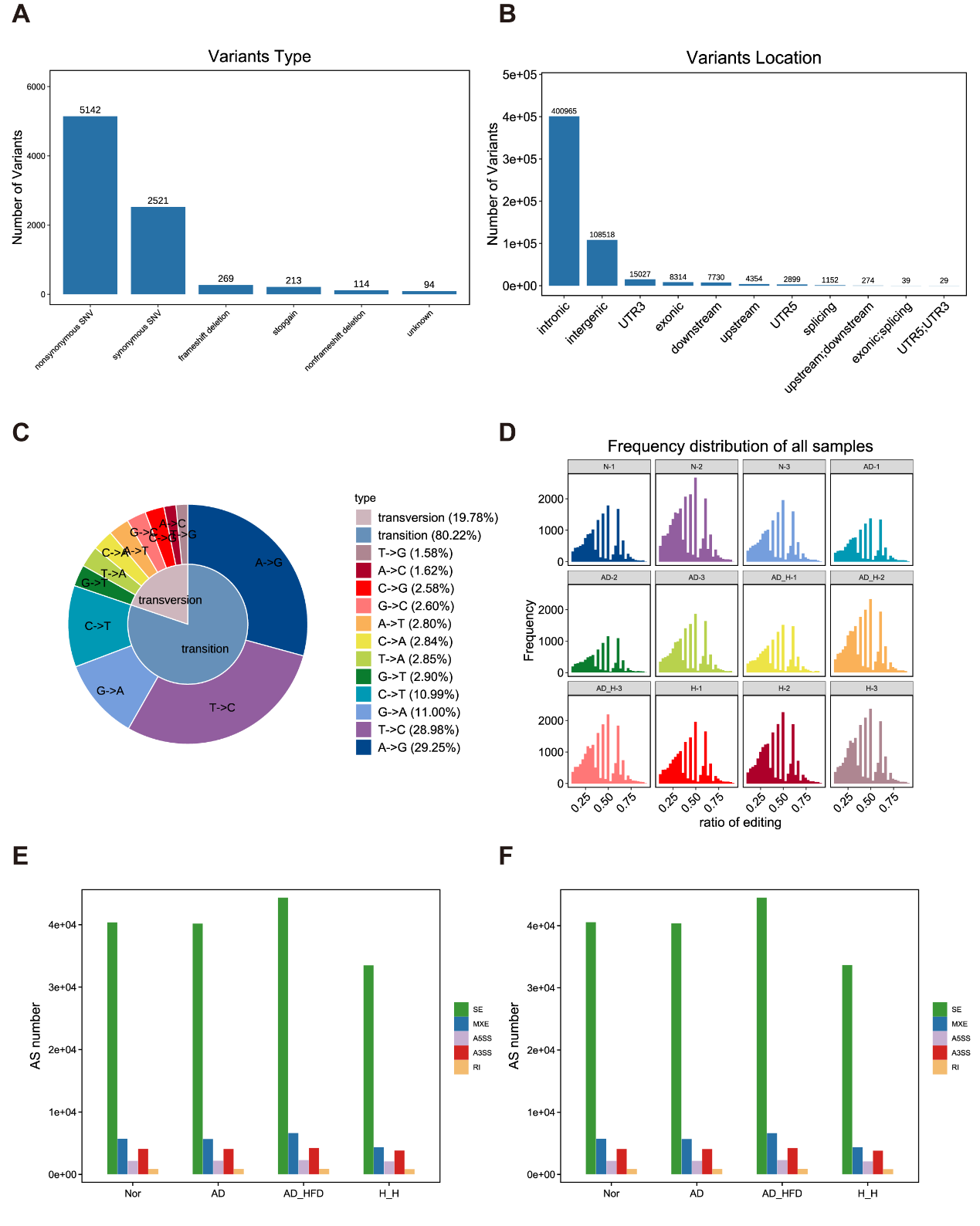


**Supplementary Figure 4** Structural analysis of transcripts.

(A) Statistical graph of SNP variant type. (B) Statistical graph of SNP variant location. (C) Statistical graph of SNP mutation type. (D) Frequency distribution of RNA editing frequency. The statistical figure of JC (E) and JCEC (F) alternative splicing.


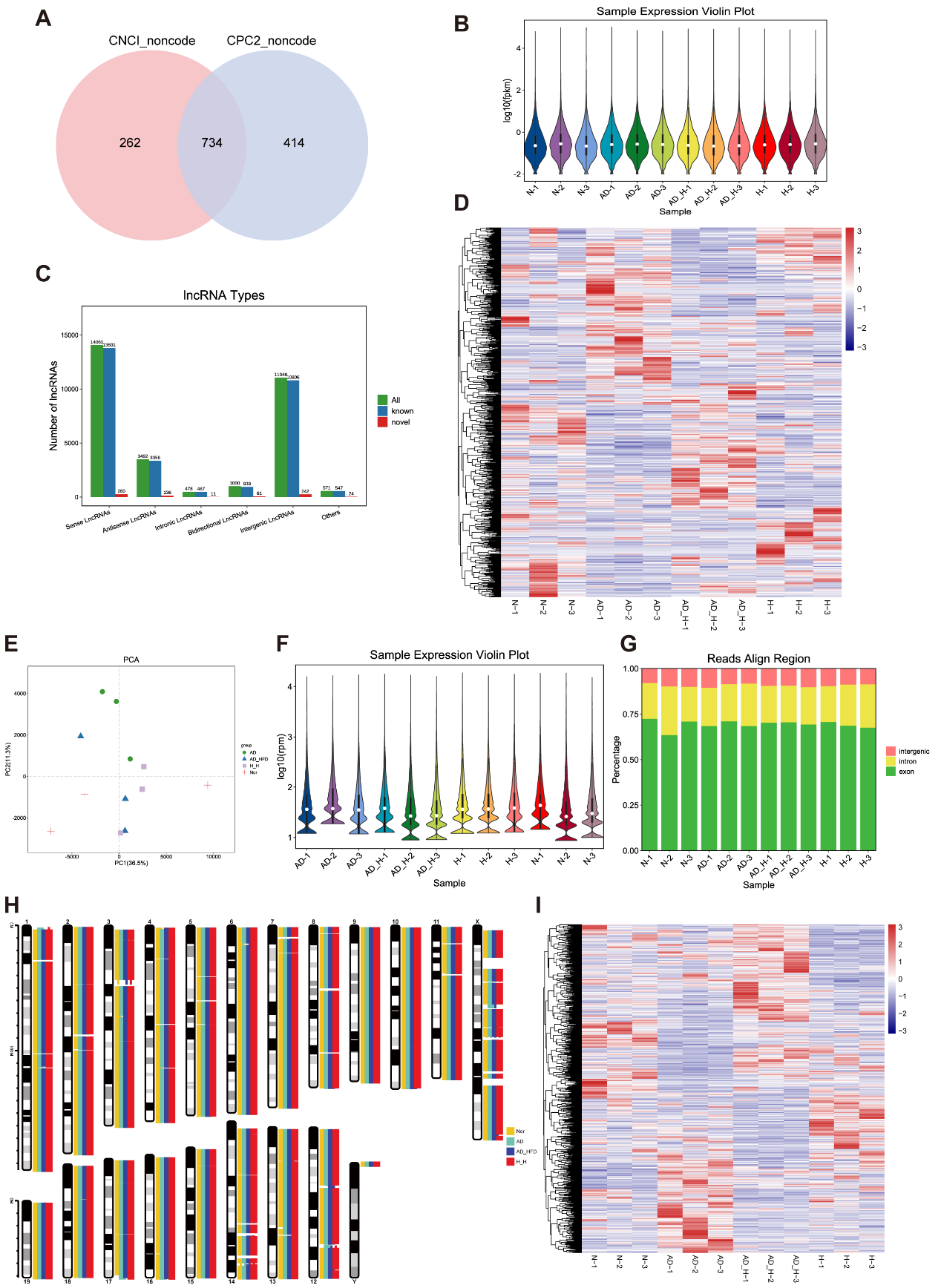


**Supplementary Figure 5** Overview of lncRNA-seq and circRNA.

(A) Venn diagram of CPC2 and CNCI. (B) Violin plot of lncRNA expression level. (C) Types of lncRNA. (D) Heatmaps of differentially expressed lncRNAs. Blue represents the lowest and red represents the highest. (E) Principal-component analysis in circRNA-seq of each sample. (F) Violin plot of circRNA expression level. (G) Reads align region in circRNA-seq. (H) Distribution of circRNAs in chromosome locations. (I) Heatmaps of differentially expressed circRNAs. Blue represents the lowest and red represents the highest. N-1, N-2 and N-3 represent Nor group biological repetition. AD-1, AD-2 and AD-3 represent AD group biological repetition. AD_H-1, AD_H-2 and AD_H-3 represent AD_HFD group biological repetition.





**Supplementary Figure 6** Original western blot for three repeats
